# Portland Cement a Material for Filling

**Published:** 1878-05

**Authors:** 

**Affiliations:** Hanover


### PORTLAND CEMENT A MATERIAL FOR FILLING.

BY DR. WITTE, HANOVER.

*Translated from Deutsche Vierteljahrsschrift fur Zahnheilkunde.*

I have for the past six months been using in my practice portland cement as a material for filling root canals, and during that period not a single case of periostitis following its use has come under my observation. The cement must be finely pulverized and kept in a well corked bottle for use. When I have a root canal to fill, I place a sufficient quantity upon a piece of glass and make it into a mass by the addition of a little creosote or carbolic acid and water. The mixture is then placed between the folds of a napkin and the excess of fluid pressed from it, by this means making it as dry as possible. The cement being thus prepared the canal must be thoroughly cleansed, the filling introduced and a piece of spunk placed upon it for few minutes to absorb further moisture. After sufficiently hard remove any particle of cement remaining in the cavity and fill with gold.

This cement unites with all the fluids remaining in the root, and forms with them in combination with the creosote an insoluble hard mass.

In so far as my experience and observation goes, this material is eminently practical, and much easier in its application than any other. Over oxy-chloride of zinc it has the advantage of being non-corrosive; over gold, of its being susceptible of a more certain adaptation, and influenced by thermal changes; over cotton in that it can not become a foul putrefactive nidus.

To-day a cadet presented himself for the purpose of having a tooth filled. Upon making an examination, I found that the cavity was so large, the walls so weak, the pulp so nearly exposed, as to render a gold filling inadvisable. Accordingly it was filled with amalgam. As soon as the operation was finished the patient was attacked with a violent intermitting pain. I removed the amalgam while yet plastic, and filled with the cement, then cutting away a portion of the cement filled again with the amalgam. The patient was discharged with the tooth at ease. Such cases are favorable to the use of the cement, and it may be employed in them I believe, with success.

